# Retinal dysfunction induced in a mouse model of unilateral common carotid artery occlusion

**DOI:** 10.7717/peerj.11665

**Published:** 2021-06-21

**Authors:** Deokho Lee, Heonuk Jeong, Yukihiro Miwa, Ari Shinojima, Yusaku Katada, Kazuo Tsubota, Toshihide Kurihara

**Affiliations:** 1 Laboratory of Photobiology, Keio University School of Medicine, Tokyo, Japan; 2 Department of Ophthalmology, Keio University School of Medicine, Tokyo, Japan; 3 Animal eye-care, Tokyo Animal Eye Clinic, Tokyo, Japan; 4 Tsubota Laboratory, Inc., Tokyo, Japan

**Keywords:** Retina, Hypoxia, Electroretinography, Ischemia, Carotid artery, Ophthalmic artery, Ocular ischemic syndrome, Stroke, Hypoperfusion, Retinal gliosis

## Abstract

**Background:**

Retinal ischemic stresses are associated with the pathogenesis of various retinal vascular diseases. To investigate pathological mechanisms of retinal ischemia, reproducible, robust and clinically significant experimental rodent models are highly needed. Previously, we established a stable murine model of chronic hypoperfusion retinal injuries by permanent unilateral common carotid artery occlusion (UCCAO) and demonstrated chronic pathological processes in the ischemic retina after the occlusion; however, retinal functional deficits and other acute retinal ischemic injuries by UCCAO still remain obscure. In this study, we attempted to examine retinal functional changes as well as acute retinal ischemic alterations such as retinal thinning, gliosis and cell death after UCCAO.

**Methods:**

Adult mice (male C57BL/6, 6–8 weeks old) were subjected to UCCAO in the right side, and retinal function was primarily measured using electroretinography for 14 days after the surgery. Furthermore, retinal thinning, gliosis and cell death were investigated using optical coherence tomography, immunohistochemistry and TUNEL assay, respectively.

**Results:**

Functional deficits in the unilateral right retina started to be seen 7 days after the occlusion. Specifically, the amplitude of b-wave dramatically decreased while that of a-wave was slightly affected. 14 days after the occlusion, the amplitudes of both waves and oscillatory potentials were significantly detected decreased in the unilateral right retina. Even though a change in retinal thickness was not dramatically observed among all the eyes, retinal gliosis and cell death in the unilateral right retina were substantially observed after UCCAO.

**Conclusions:**

Along with previous retinal ischemic results in this model, UCCAO can stimulate retinal ischemia leading to functional, morphological and molecular changes in the retina. This model can be useful for the investigation of pathological mechanisms for human ischemic retinopathies and furthermore can be utilized to test new drugs for various ischemic ocular diseases.

## Introduction

Retinal ischemia is a general cause of visual impairment and blindness all over the world (Osborne et al., 2004). It is associated with a huge range of clinical ocular disorders and diseases such as diabetic retinopathy, glaucoma, optic neuropathies and stroke (Dattilo, Newman & Biousse, 2018; Fouda et al., 2020; Minhas, Morishita & Anand, 2012; Osborne et al., 2004). As oxygen (directly referred to as “blood”) in the retina is supplied through the ophthalmic artery originated from the internal carotid artery, one of two branches of the common carotid artery (CCA), animal models by common carotid artery occlusion or stenosis (CCAO or CCAS) have been used to understand the pathogenesis of retinal ischemia (Crespo-Garcia et al., 2018; Davidson et al., 2000; Lavinsky et al., 2006; Lee et al., 2020b; Qin et al., 2019; Sun et al., 2019; Venkat, Chopp & Chen, 2015; Wellons Iii et al., 2000; Yamamoto et al., 2006; Yang et al., 1997).

In human, CCAO or CCAS is a devastating cause of ocular ischemic syndrome (Faries et al., 2006). Ocular ischemia resulting from CCAO or CCAS has gradually received attention in that several ocular disorders, such as ischemic optic neuropathy, diabetic retinopathy and glaucoma, are suggested to be involved with cardiovascular diseases-associated retinal ischemic states (Chou et al., 2018; Drinkwater, Davis & Davis, 2020; Harris et al., 1999; Osborne et al., 2004). Therefore, CCAO or CCAS has been attempted to develop animal models for such retinal ischemic diseases. However, several previous studies demonstrated that experimental models (mice and rats) could die during and after CCAO or CCAS, in that severe brain damages as well as cardiac arrests were induced by the operation (Crespo-Garcia et al., 2018; Venkat, Chopp & Chen, 2015; Wellons Iii et al., 2000; Yang et al., 1997). This evokes several matters such as unexpected loss and individual variations of experimental models. Furthermore, severe brain injuries have changes to cause retinal damages directly (termed as “retrograde degeneration”) (Venkat, Chopp & Chen, 2015), not by retinal ischemia itself.

In this regard, we previously established a stable mouse model of retinal ischemia induced by unilateral CCAO (UCCAO) with 100% survival rate during and after the operation through minimization of brain damages and showed time courses of common ischemic findings in the unilateral retina (Lee et al., 2020a), which were generally seen in other CCAO and CCAS models (Crespo-Garcia et al., 2018; Davidson et al., 2000; Lavinsky et al., 2006; Lee et al., 2020b; Ogishima et al., 2011; Qin et al., 2019; Sun et al., 2019; Venkat, Chopp & Chen, 2015; Wellons Iii et al., 2000; Yamamoto et al., 2006; Yang et al., 1997). However, time courses of retinal functional changes and acute retinal injuries in this model have not been clearly studied. In this current study, we attempted to evaluate time-dependent changes in retinal function after UCCAO using electroretinography (ERG). Moreover, we investigated acute alterations by retinal ischemia such as retinal thinning, gliosis and cell death after UCCAO with time courses using optical coherence tomography (OCT), immunohistochemistry and TUNEL assay, respectively.

## Materials & Methods

### Animal

Adult mice (male C57BL6, 6–8 weeks old, *n* = 42) were purchased from CLEA Japan (Tokyo, Japan) and they were maintained in a temperature (24 ± 1 °C)-controlled environment (5–6 mice per cage) under a 12 h light-dark cycle. They were given ad libitum access to food and water. Any sign of diseases were constantly checked every day. All animal experimental protocols were approved by Keio University School of Medicine IACUC (Institutional Animal Care and Use Committees, approved number: #16017), and all experiments were carried out in accordance with the international standards of animal care and use in the ARVO (Association for Research in Vision and Ophthalmology) Statement for the Use of Animals in Ophthalmic and Vision Research.

### Unilateral common carotid artery occlusion (UCCAO)

UCCAO was conducted as previously described (Lee et al., 2020a) with a minor modification regarding an anesthetic technique. We assigned 21 mice for UCCAO and age-matched 21 mice for a sham surgery. After randomization of 42 mice without any special restrictions, they were individually anesthetized by intraperitoneal injection with a combination of MMB (midazolam, medetomidine and butorphanol tartrate) as previously described (Miwa, Tsubota & Kurihara, 2019b; Tomita et al., 2020). After anesthesia, the right CCA was occluded clearly by two 6–0 silk sutures and cut between the two ties by a surgical scissor. After the operation, mice were recovered quickly by intraperitoneal injection with a solution of atipamezole hydrochloride as previously described (Miwa, Tsubota & Kurihara, 2019b; Tomita et al., 2019). For the management of pain, 0.4 mg/kg of butorphanol tartrate was injected to mice when mice woke up (Lee et al., 2020b). For a sham surgery, mice underwent the same step except for the occlusion. At the end of all the experiments or during the experiments in which signs such as hunched posture, lethargy, lack of food intake or infection of the surgery site were seen in mice, a combination of 3x of MMB was injected to mice (Lee et al., 2020b) and then mice were euthanized under deep anesthesia.

### Electroretinography (ERG)

ERG was set and conducted using a Ganzfeld dome, PuREC acquisition system and LED stimulators by a blinded investigator as previously described (Miwa, Tsubota & Kurihara, 2019b; Tomita et al., 2019). Following dark adaptation more than 12 h, mice were anesthetized by intraperitoneal injection with a combination of MMB under dim red light in a dark room. The pupils were dilated by a solution of 0.5% tropicamide and 0.5% phenylephrine. The active electrodes were touched on contact lens and the reference electrode was inserted into the mouth. Scotopic responses were recorded with various intensities of stimuli under dim red light in a dark room. Heat pads were prepared to keep all mice warm during the procedure. The amplitude of a-wave was calculated from the baseline to the lowest point of a-wave and the amplitude of b-wave was calculated from the lowest point of a-wave to the peak of b-wave. The amplitudes of oscillatory potentials (OPs) were measured at the four peaks of OPs as previously described (Tomita et al., 2020). When the electrodes were detached from contact lens of mice by unexpected movements of the electrodes during the experiments, data points were marked and excluded for further analyses. Except for this issue, all data points from the experiments were included for further analyses. Confounders were not controlled as experiments were processed randomly and individually.

### Immunohistochemistry

The eyes fixed in 4% PFA-added tubes for 24 h were incubated with 10–30% sucrose on ice until the eyes dropped to the bottom of the tubes. O.C.T. Compound (Sakura Tissue-Tek, Tokyo, Japan) was used to quickly embed the eyes for frozen sectioning. Cryostat (Leica CM3050S, Leica, Wetzlar, Germany) was conducted to obtain sagittal sectioning slides of the eyes. The sagittal sectioning slides were washed with PBS three times and then incubated in blocking solution (PBS + 0.1% Triton + 0.1% BSA) for 1 h at room temperature. The retinas were incubated with a primary antibody (GFAP 1:400, Cat #13-0300, Thermo Fisher Scientific, USA) overnight at 4 °C. The retinas were washed with PBS + 0.1% Triton several times and incubated with a species-appropriate fluorescence-conjugated secondary antibody (Thermo Fisher Scientific, USA) for 2 h at room temperature in a dark room. After washing with PBS + 0.1% Triton three times, DAPI was incubated for 1 min in order to stain nuclei. After washing with PBS three times, the retinas were mounted and examined via a fluorescence microscope (LSM710, Carl Zeiss, Jena, Germany), as previously described (Lee et al., 2020b). The fluorescence immunoreactivity was quantified by a morphology score as previously described (Lee et al., 2020a; Yamamoto et al., 2006) with a minor modification: 0 = no signal, 1 = labelled processes in the ganglion cell layer, 2 = labelled processes in the inner retinal layer including the ganglion cell layer, and 3 = labelled processes in the entire retinal layer including the inner and outer retinas.

### TUNEL assay

Sagittal sectioning slides of the eyes were subjected to TUNEL assay. The assay was conducted following the manufacturer’s instruction (in situ Apoptosis Detection Kit, Cat #MK500, Takara Bio, Japan). Briefly, the slides were washed with PBS three times. Permeabilization buffer was applied to the slides for 5 min on ice. After washing with PBS three times, labeling reaction mixture containing TdT enzymes were applied to the samples for 1 h. After washing with PBS three times, DAPI was added for 1 min to stain nuclei. After washing with PBS three times, the slides were mounted and examined via a fluorescence microscope (LSM710; Carl Zeiss, Jena, Germany).

### Optical coherence tomography (OCT)

We performed OCT (Envisu R4310, Leica, Wetzlar, Germany) as previously described (Miwa et al., 2019a). Briefly, a combination of 0.5% tropicamide and 0.5% phenylephrine (Santen Pharmaceutical, Osaka, Japan) was added to mice for 5 min for induction of mydriasis. Anesthetized mice by a combination of MMB were placed in an OCT platform and then OCT was immediately conducted. 0.2, 0.4 and 0.6 mm from the optic nerve head in the retina were marked. Then, total retinal thickness (from the outer retina to the inner retina including the ganglion cell layer) was measured via OCT software (Envisu R4310, Leica, Wetzlar, Germany).

### Statistical analysis

Analyses of data from the experiments were conducted with GraphPad Prism 5 (USA). All values in data were presented as mean ± standard deviation. Statistical significance was calculated by using a two-tailed Student’s *t*-test or a two-way ANOVA followed by a Bonferroni post hoc test, and *p*-value of less than 0.05 was considered to be significant statistically.

## Results

### Decreases in the amplitudes of a- and b-waves after UCCAO

**Figure 1:**
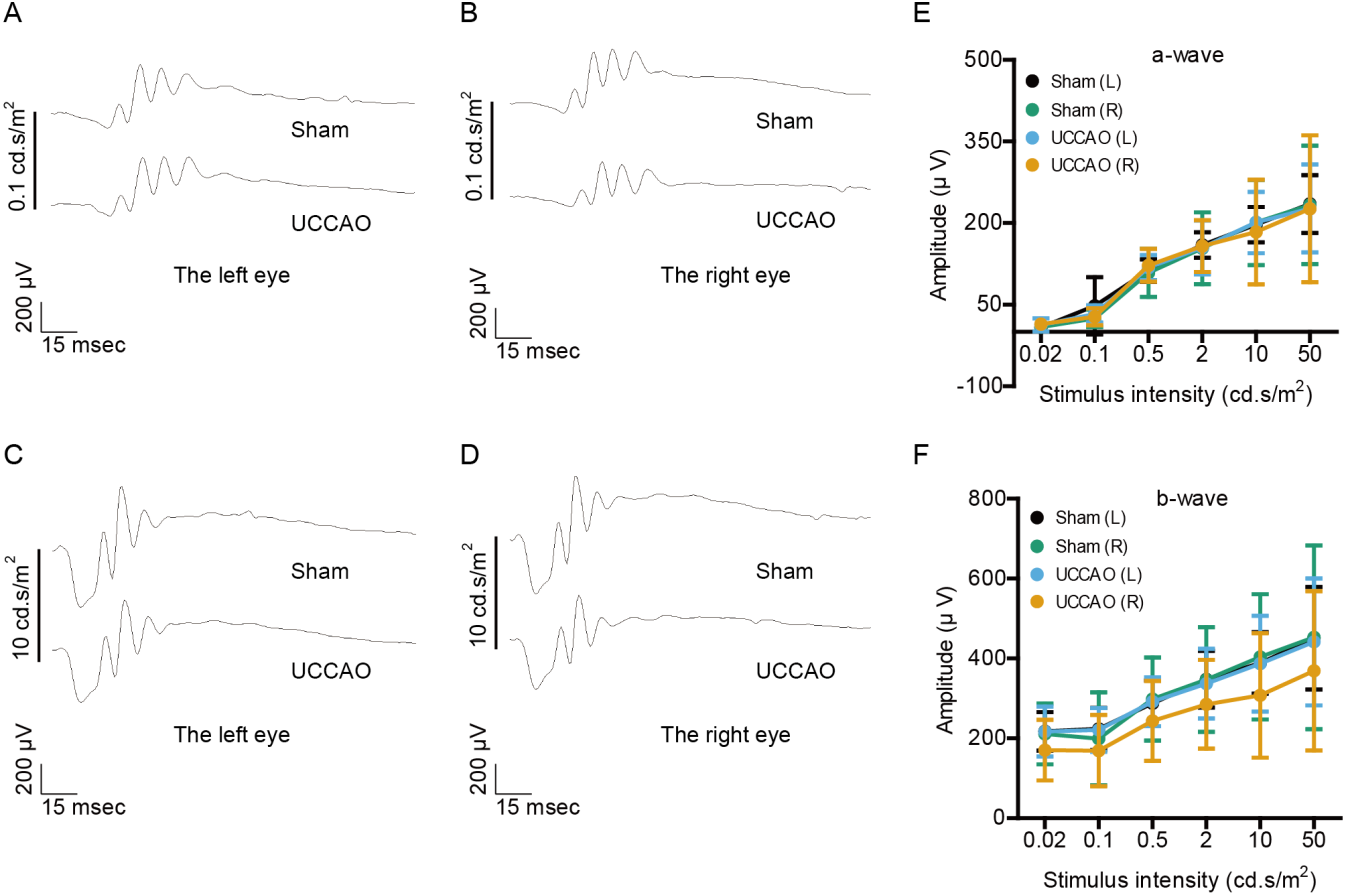
Any change in retinal function is not seen on day 3 after UCCAO. (A–D) Representative waveforms of a- and b-waves (low and high intensities, 0.1 and 10 cd s/m^2^, respectively) of the right and left eyes in the sham- and UCCAO-operated mice (*n* = 8 for UCCAO-operated group and 8 for sham-operated group) and (E–F) quantitative analyses showed that the amplitudes of a- and b-waves were not significantly different among all of the eyes in sham- and UCCAO-operated mice 3 days after the operation. Graphs were presented as mean ±  standard deviation. The data were analyzed using a two-tailed Student’s *t*-test. *P* > 0.05. Black (sham L), the sham-operated left eye; bluish green (sham R), the sham-operated right eye; sky blue (UCCAO L), the UCCAO-operated left eye; orange (UCCAO R), the UCCAO-operated right eye.

We examined whether retinal function could be altered 3 days after UCCAO (Fig. 1). The amplitudes of a- and b-waves were not significantly different between the UCCAO-operated mice and the sham-operated mice. There was an only slight decrease in the amplitude of b-wave in the UCCAO-operated right eye in comparison with that of b-wave in the sham-operated right eye.

**Figure 2:**
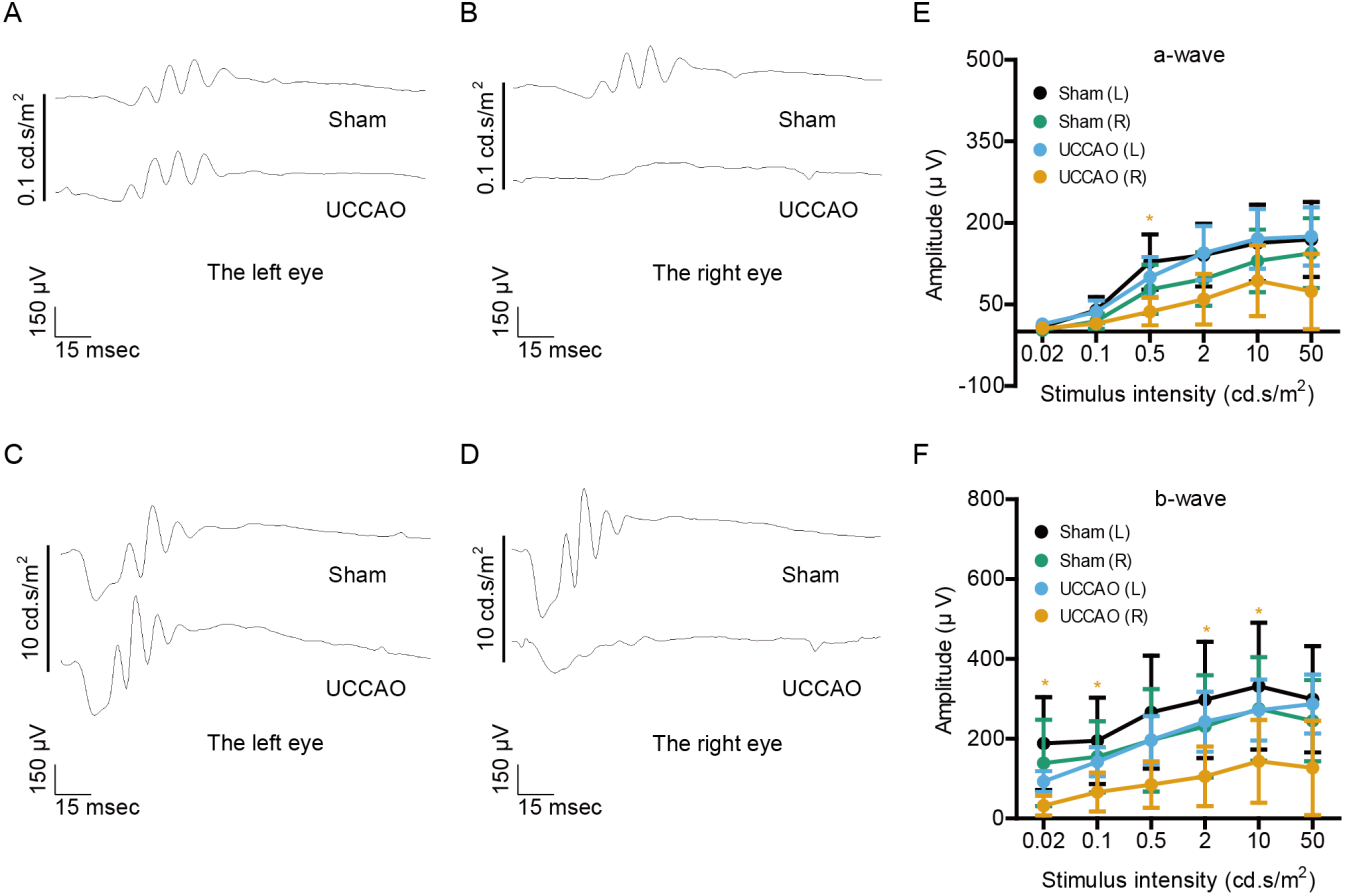
Changes in retinal function are seen on day 7 after UCCAO. (A–D) Representative waveforms of a- and b-waves (low and high intensities, 0.1 and 10 cd s/m^2^, respectively) of the right and left eyes in sham- and UCCAO-operated mice (*n* = 8 for UCCAO-operated group and 8 for sham-operated group) and (E–F) quantitative analyses showed that the amplitude of b-wave dramatically decreased in the UCCAO-operated unilateral right eye, in comparison with that in the sham-operated right eye 7 days after the operation. Graphs were presented as mean ±  standard deviation. The data were analyzed using a two-tailed Student’s *t*-test. * *P* < 0.05. Black (sham L), the sham-operated left eye; bluish green (sham R), the sham-operated right eye; sky blue (UCCAO L), the UCCAO-operated left eye; orange (UCCAO R), the UCCAO-operated right eye.

Next, retinal function was further examined 7 days after UCCAO (Fig. 2). We found a significant decrease in the amplitude of b-wave in the UCCAO-operated right eye, in comparison with that of b-wave in the sham-operated right eye. There was a decreasing tendency with significance in the amplitude of a-wave in the UCCAO-operated right eye, in comparison with that of a-wave in the sham-operated right eye. When it comes to the left eye, we could not find a significant alteration in retinal function after UCCAO.

**Figure 3:**
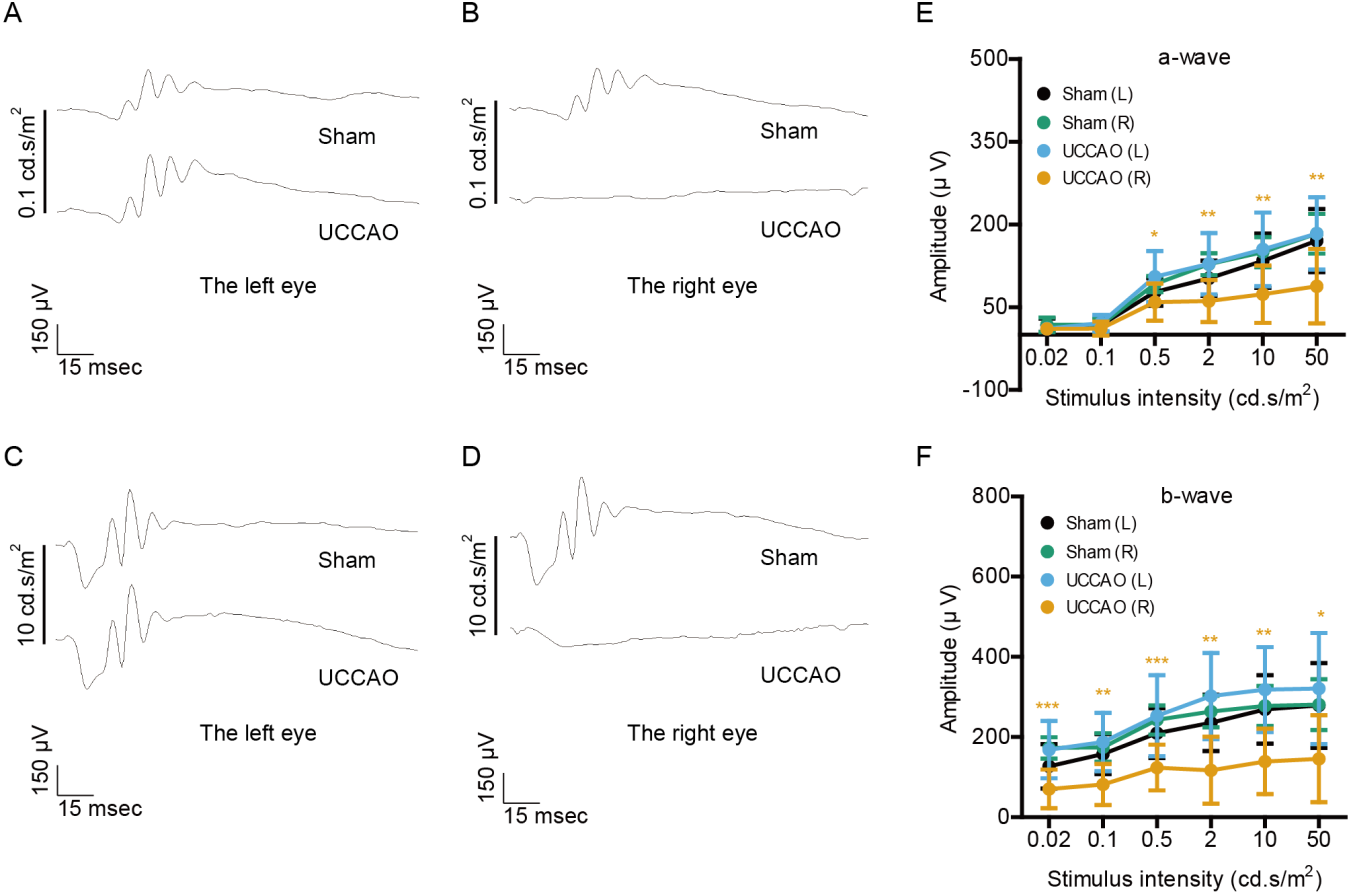
Changes in retinal function are clearly seen on day 14 after UCCAO. (A–D) Representative waveforms of a- and b-waves (low and high intensities, 0.1 and 10 cd s/m^2^, respectively) of the right and left eyes in sham- and UCCAO-operated mice (*n* = 8 for UCCAO-operated group and 8 for sham-operated group) and (E–F) quantitative analyses showed that the amplitudes of a- and b-waves significantly and dramatically decreased in the UCCAO-operated unilateral right eye, in comparison with those in the sham-operated right eye 14 days after the operation. Graphs were presented as mean ±  standard deviation. The data were analyzed using a two-tailed Student’s *t*-test. * *P* < 0.05, ** *P* < 0.01, *** *P* < 0.001. Black (sham L), the sham-operated left eye; bluish green (sham R), the sham-operated right eye; sky blue (UCCAO L), the UCCAO-operated left eye; orange (UCCAO R), the UCCAO-operated right eye.

On day 14 after UCCAO, we could more clearly see a dramatic decrease in the amplitudes of both a- and b-waves in the UCCAO-operated right eye, in comparison with those of waves in the sham-operated right eye (Fig. 3). We still could not find a significant alteration in left retinal function after UCCAO.

### Decreases in the amplitudes of OPs after UCCAO

**Figure 4:**
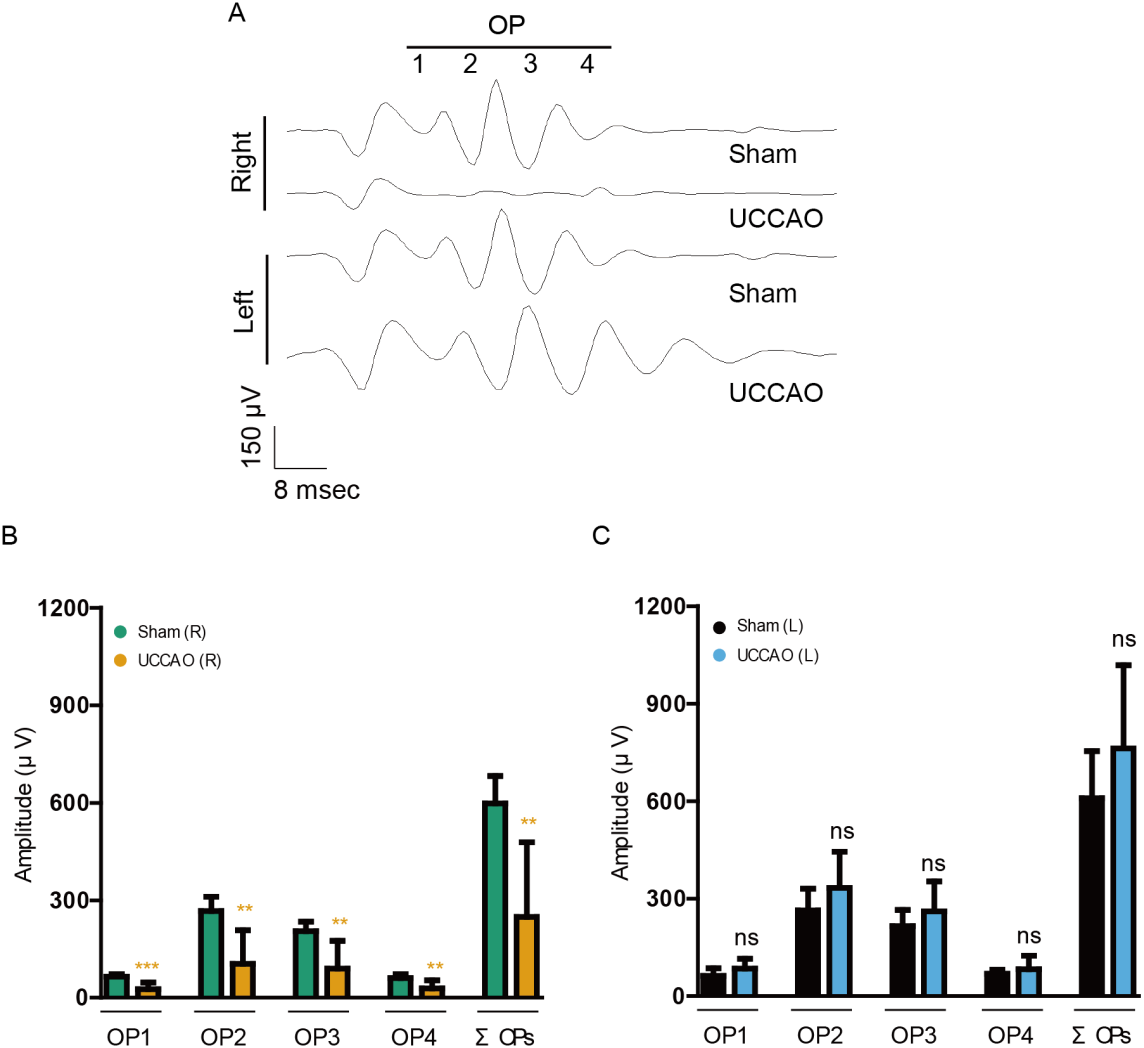
A reduction in the amplitudes of OPs is seen on day 14 after UCCAO. (A) Representative waveforms of OPs of the right and left eyes in the sham- and UCCAO-operated mice (*n* = 8 for UCCAO-operated group and 8 for sham-operated group) and (B–C) quantitative analyses showed that the amplitudes of OPs significantly decreased in the UCCAO-operated unilateral right eye, in comparison with those in the sham-operated eye 14 days after the operation. Graphs were presented as mean ± standard deviation. The data were analyzed using Student’s *t*-test. ** *P* < 0.01, *** *P* < 0.001. Black (sham L), the sham-operated left eye; bluish green (sham R), the sham-operated right eye; sky blue (UCCAO L), the UCCAO-operated left eye; orange (UCCAO R), the UCCAO-operated right eye. OP, oscillatory potential.

As retinal dysfunction in the right eye was observable clearly on day 14 after UCCAO, we further examined retinal dysfunction using another value, “OPs” (Fig. 4). The reduced amplitudes of OPs are known to occur in various types of murine and human retinopathies (Crespo-Garcia et al., 2018; Davidson et al., 2000; Hancock & Kraft, 2004; Kizawa et al., 2006; Kurihara et al., 2008; Li et al., 2002; Qin et al., 2019; Shirao & Kawasaki, 1998; Tomita et al., 2019). Reduction in OP1, OP2, OP3, OP4 and total OPs (ΣOPs) amplitudes was observed in the UCCAO-operated right eye, in comparison with that in the sham-operated right eye. There was no significant difference in the amplitudes between the sham and UCCAO-operated left eyes.

### A transient decrease in the amplitude ratio of b/a wave after UCCAO

**Figure 5:**
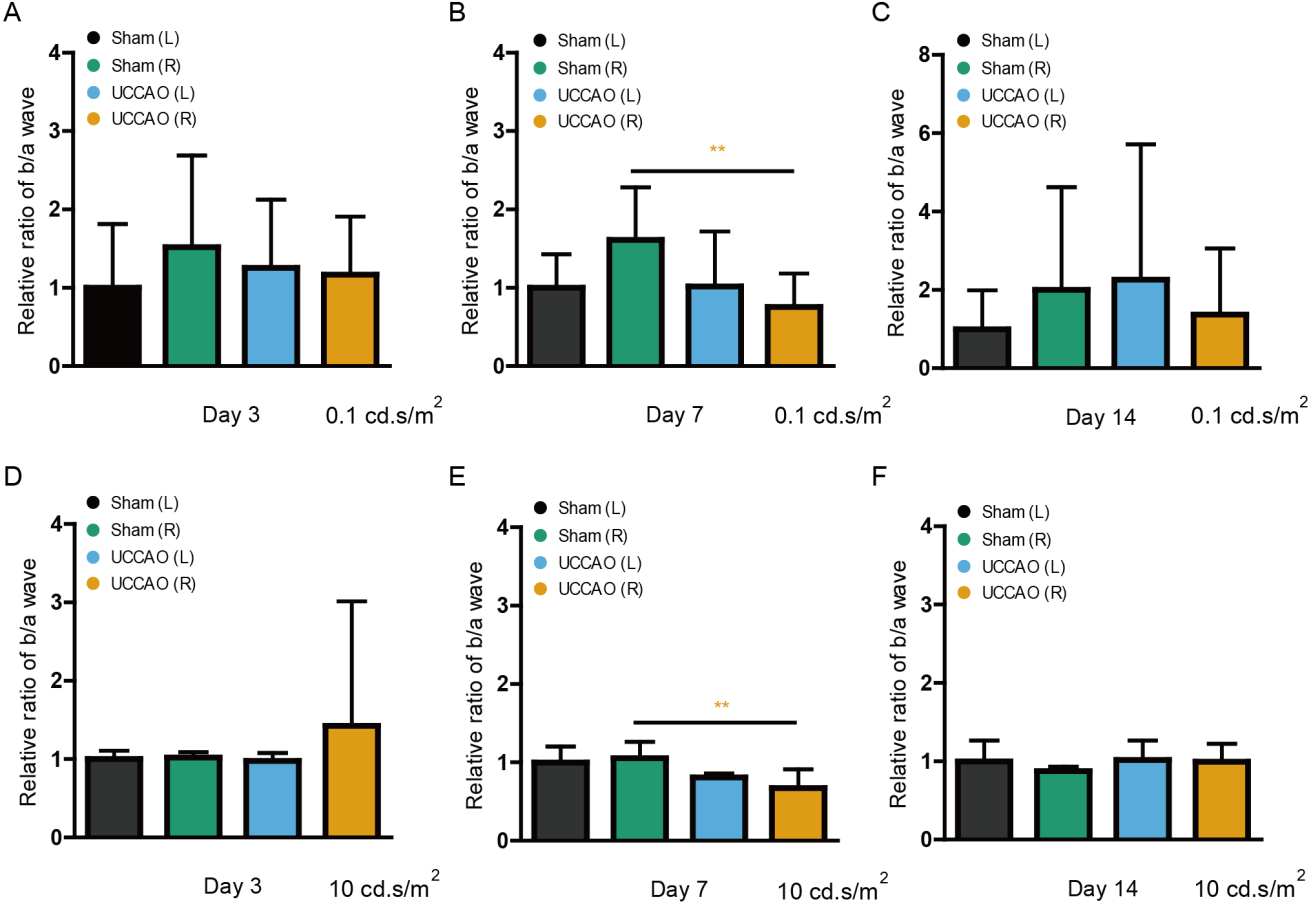
A transient reduction in the amplitude ratio of b/a wave is seen after UCCAO. (A–F) Quantitative analyses based on the values of the amplitude ratio of b/a wave from Fig. 1 to Fig. 3 (low and high intensities, 0.1 and 10 cd s/m^2^, respectively) showed that its ratio significantly decreased in the UCCAO-operated unilateral right eye, in comparison with that in the sham-operated eye only on day 7 after the operation. Graphs were presented as mean ±  standard deviation. The data were analyzed using Student’s *t*-test. ** *P* < 0.01. Black (sham L), the sham-operated left eye; bluish green (sham R), the sham-operated right eye; sky blue (UCCAO L), the UCCAO-operated left eye; orange (UCCAO R), the UCCAO-operated right eye.

After ERG analyses, we retrospectively calculated the b/a wave amplitude ratio (low and high intensities: 0.1 and 10 cd s/m^2^) on day 3, 7 and 14 (Fig. 5). The significantly decreased ratio in the UCCAO-operated right eye was seen on day 7 after UCCAO. The decreased b/a wave amplitude ratio returned to the range similar to the range in the sham-operated eye on day 14 after UCCAO.

### Observation in retinal thickness changes after UCCAO

**Figure 6:**
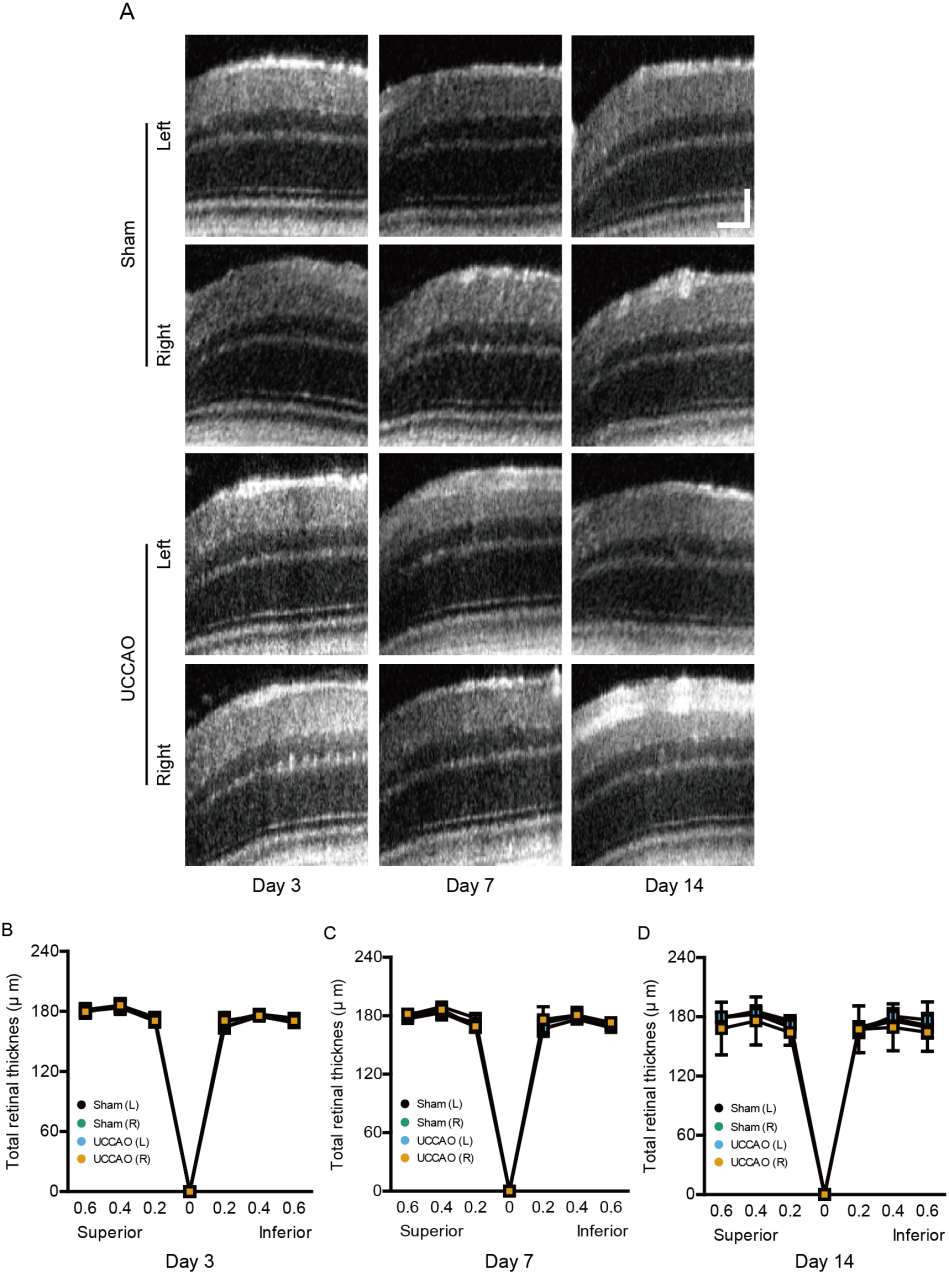
A dramatic change in retinal thickness is not detected for 14 days after UCCAO. (A) Representative OCT images in the sham- and UCCAO-operated retinas and (B–D) quantitative analyses showed that there was no change in retinal thickness in the right and left eyes in the sham- and UCCAO-operated mice (*n* = 6–7 for UCCAO-operated group and 6–7 for sham-operated group) for 14 days after the operation. Horizontal scale bar: 100 µm; Vertical scale bar: 50 µm. *P* > 0.05. The values in the horizontal axis of spider diagrams represent 0.2, 0.4 and 0.6 mm distant from the optic nerve head (0). The measurement site in the representative example images was 400 µm from the optic nerve head. The data were analyzed using two-way ANOVA followed by a Bonferroni post hoc test. Spider diagrams were presented as mean with ±  standard deviation. Black (sham L), the sham-operated left eye; bluish green (sham R), the sham-operated right eye; sky blue (UCCAO L), the UCCAO-operated left eye; orange (UCCAO R), the UCCAO-operated right eye.

We further examined whether retinal thickness could be changed by UCCAO. Although there was slight fluctuation in retinal thickness for 14 days after UCCAO, we could not detect a significant change in total retinal thickness for 14 days after UCCAO (Fig. 6). During the experimental period, only one mouse showed a severe cataract; therefore retinal thickness in this mouse could not be measured by OCT.

### Retinal gliosis and cell death after UCCAO

**Figure 7:**
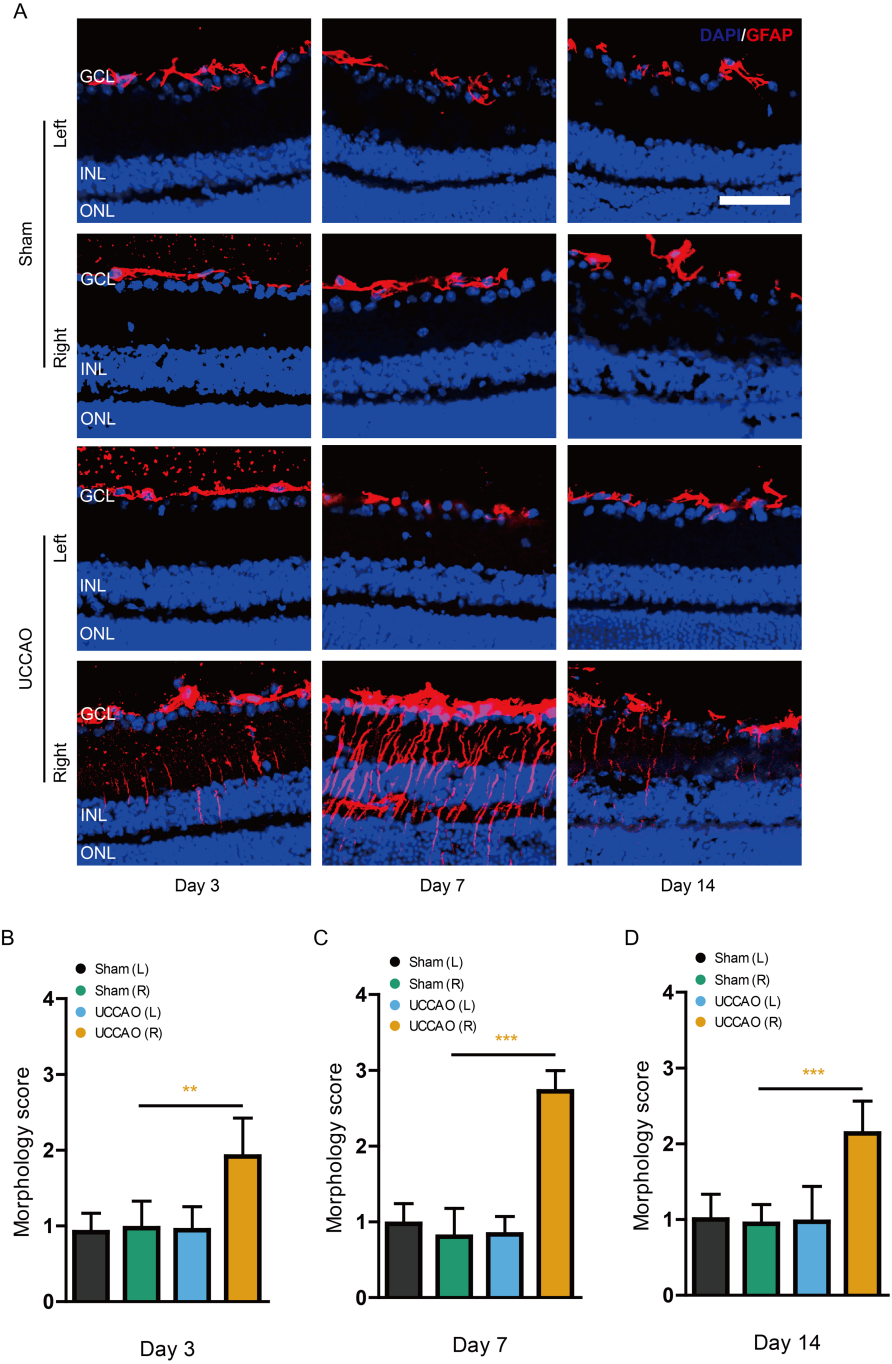
Retinal gliosis is continuously observed for 14 days after UCCAO. (A) Representative images and (B–D) quantitative analyses showed that there was retinal gliosis stained by GFAP in the right retina in the UCCAO-operated mice (*n* = 6 for UCCAO-operated group and 6 for sham-operated group) for 14 days after the operation. Scale bar: 50 µm. Graphs were presented as mean with ±standard deviation. The data were analyzed using Student’s *t*-test. ** *P* < 0.01, *** *P* < 0.001. Black (sham L), the sham-operated left eye; bluish green (sham R), the sham-operated right eye; sky blue (UCCAO L), the UCCAO-operated left eye; orange (UCCAO R), the UCCAO-operated right eye. GCL, ganglion cell layer; INL, inner retinal layer; ONL, outer retinal layer.

**Figure 8:**
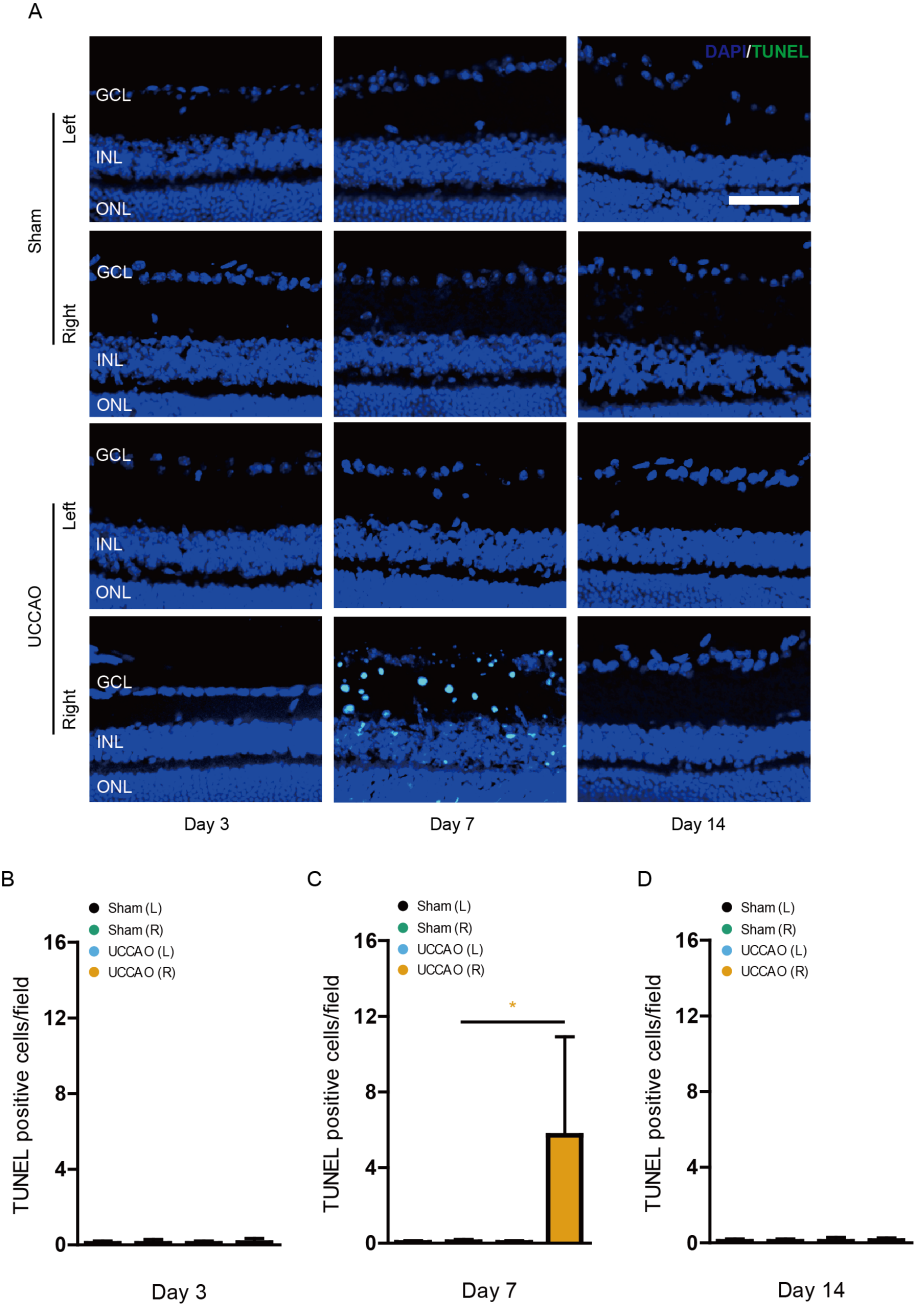
Retinal cell death is observed on day 7 after UCCAO. (A) Representative images and (B-D) quantitative analyses showed that there were TUNEL (+) cells in the right retina in the UCCAO-operated mice (*n* = 6 for UCCAO-operated group and 6 for sham-operated group) only 7 days after the operation. Scale bar: 50 µm. Graphs were presented as mean with ±standard deviation. The data were analyzed using Student’s *t*-test. * *P* < 0.05. Black (sham L): the sham-operated left eye; bluish green (sham R): the sham-operated right eye; sky blue (UCCAO L): the UCCAO-operated left eye; orange (UCCAO R): the UCCAO-operated right eye. GCL: ganglion cell layer; INL: inner retinal layer; ONL: outer retinal layer.

We examined whether retinal gliosis and retinal cell death could be induced by UCCAO. Acute retinal gliosis has been reported in this model (Lee et al., 2020a). We could also detect this finding on day 3, analyzed by a higher morphology score in the right retina after UCCAO, compared with that in the sham-operated right retina (Fig. 7). This finding was maintained for 14 days after UCCAO (Fig. 7). When it comes to retinal cell death, even though we could not detect TUNEL (+) cells in the retina on day 3 and 14, TUNEL (+) cells were substantially detected in the right retina on day 7 after UCCAO (Fig. 8).

## Discussion

The present study was designed to evaluate whether UCCAO-induced ischemic stresses in the retina could contribute to the development of retinal dysfunction and acute retinal injuries. Based on previous data, UCCAO has been suggested to cause abnormalities in retinal blood flow, stabilization of hypoxia-inducible factor-1 *α* (HIF-1 *α*) and HIF-2 *α* in the unilateral retina, induction of hypoxia-responsive genes including hypoxia-induced inflammatory cytokines, acute retinal reactive gliosis and chronic inner retinal thinning (Lee et al., 2019; Lee et al., 2020a; Lee et al., 2021). Although several retinal ischemic findings were unraveled in this model, there were missing points, especially retinal functional changes and acute retinal injuries. In this current study, we firstly demonstrated functional impairment in the retina could gradually occur from 3 days to 7 days after the occlusion and its impairment could be clearly observed 14 days after the occlusion. Furthermore, its functional deficit could be explained with acute retinal cell death as well as continuous retinal gliosis by the occlusion, which is a significance of our study.

ERG recordings were used in order to evaluate retinal functional changes by UCCAO. a-wave provides functional status of photoreceptors (Brown, 1968; Robson et al., 2003), whereas b-wave provides conditions on functional activities of Müller cells and/or bipolar cells (Miller & Dowling, 1970). Moreover, cellular origins of OPs are neurons in the inner retinal layer such as bipolar and amacrine cells (Heynen, Wachtmeister & Van Norren, 1985; Wachtmeister, 1998; Yonemura & Kawasaki, 1979). We found that the amplitude of b-wave was more affected than that of a-wave by UCCAO, and the amplitudes of OPs dramatically became lower. In our previous data and present data, reactive gliosis in Müller cells and astrocytes, which is activated under ocular ischemic conditions (Vecino et al., 2016), was mostly detected in the inner retina layer among the whole retinal layers after UCCAO (Lee et al., 2020a). Retinal cell death was more observable in the inner retina 7 days after UCCAO. Even though there was no acute change in retinal thickness, inner retinal thinning was observed more than the outer retinal thinning after UCCAO at the chronic stage, 10 weeks after UCCAO (Lee et al., 2020a). A pathological change in other CCAO and CCAS models was also a decrease in thickness of the inner retinal layer (Crespo-Garcia et al., 2018; Kalesnykas et al., 2008; Lavinsky et al., 2006; Ogishima et al., 2011; Qin et al., 2019; Sun et al., 2019; Yamamoto et al., 2006). With this together, carotid artery occlusion-induced retinal ischemic models seem applicable to study pathological mechanisms of functional and histological ischemic damages in the inner retinal layer at the chronic stage. However, UCCAO also damages the outer retinal layer, analyzed by retinal gliosis in the entire retinal layer, retinal cell death in the outer retina and a reduction in the amplitude of a-wave after UCCAO with a transient decrease in the amplitude ratio of b/a wave. This can be explained by the fact that vessels supplying the outer retina and the inner retina are originated from the same internal carotid artery, one of two branches of the common carotid artery. Anatomical ocular circulation is needed to be taken into account for deep understanding of inner and outer retinal ischemic damages by UCCAO. Therefore, a comparison analysis on damaging intensities between the outer and the inner retinas along with alterations in ocular circulation (choroid and retinal circulation) by UCCAO will be further studied.

Even though this mouse model shows common retinal ischemic injuries, several limitations for interpretation of the current outcomes exist. Compared to murine models of bilateral common carotid artery occlusion and stenosis (Kalesnykas et al., 2008; Lavinsky et al., 2006; Qin et al., 2019; Sun et al., 2019; Yamamoto et al., 2006), milder retinal ischemic stress is observed in this UCCAO model. In this regard, retinal thinning was not dramatically observed in a short-term period even though there was retinal cell death. In fact, retinal cell death in this UCCAO model is also seen weaker than that in other retinal injury models (Luo et al., 2020; Matei et al., 2020; Song et al., 2008). Long-term period studies may be necessary to observe histological injuries in the retina by UCCAO. However, this milder effect could be more relevant to study ischemic retinopathies in human, in that retinal ischemic injuries in human are gradually induced with time (Terelak-Borys, Skonieczna & Grabska-Liberek, 2012). Even though our current data showed retinal cell death in the outer and inner retina, what kinds of cells are damaged after UCCAO has not been deeply studied. Therefore, co-labeling of TUNEL with specific retinal cell markers is needed to expend understanding of retinal cell death depending on the cell types by UCCAO, which will be further studied. Next, metabolic complications have been closely associated with the development of human ischemic retinopathies such as ocular ischemic syndrome or diabetic retinopathies (Sim et al., 2013). Such metabolic complications have not been applied to this model in this study. Therefore, the ischemic processes from this model may not be directly corresponded with the development of human ischemic retinopathies. Nonetheless, this issue could be solved with combination of murine models of metabolic diseases such as diabetes (genetically diabetic db/db mice and/or streptozotocin-induced diabetic mice) with UCCAO. With this approach, we can develop more clinically relevant murine models of ischemic retinopathies. Next, we selected MMB (midazolam, medetomidine and butorphanol tartrate) for the surgery as well as OCT and ERG recordings. MMB has been widely used as a favorable anesthetic in experimental models since ketamine was enrolled as a narcotic drug in Japan in 2007 (Kawai et al., 2011; Kirihara et al., 2019). Even though all mice received the same anesthetic procedure in this study, it may be important to deeply consider appropriate anesthetics and time of anesthesia for the surgery as well as OCT and ERG recordings.

In our current study, we found that only the right (ipsilateral) eye was dramatically damaged by UCCAO. When it comes to the left (contralateral) eye, there was no significant difference in the retinal ischemic outcomes between the sham- and UCCAO-operated mice. As the left retina can be considered as a control for the right retina, we can minimize the number of experimental animals for further other studies such as *in vivo* drug screenings or knock-out mouse work which needs substantial handling and breeding of mice. Moreover, UCCAO model can reduce inter-individual differences. Substantial inter-individual variabilities are not evitable even though genetically identical experimental mice are used for experimental studies (Freund et al., 2013; Van Dongen et al., 2012). In this regard, this UCCAO model has a strong benefit for comparison analyses by using left and right eyes in the same individual animals. This implies a reduction in variations of retinal and circulatory conditions of each animal and other variable factor such as diet and oxygen consumption. However, more studies may be required for using the left (contralateral) retina as a control in that unexpected compensation signals from the right (ipsilateral) ischemic retinal damages (which we have not found yet) might occur in the left (contralateral) retina.

## Conclusions

In conclusion, along with previous findings in this model (Lee et al., 2019; Lee et al., 2020a; Lee et al., 2021), we have demonstrated exact time courses of pathological dysfunction, continuous reactive gliosis and transient cell death in the unilateral retina after UCCAO in mice. After UCCAO, retinal blood flow becomes abnormal, leading to retinal ischemia confirmed by stabilization of HIF-1 *α* and HIF-2 *α* with induction of hypoxia-responsive genes and hypoxia-induced inflammatory cytokines. Next, acute reactive gliosis and cell death in the retina occurs and retinal function decreases at the early stage. Finally, the inner retinal layer becomes thinner with reduction in inner retinal cell maker gene expressions at the chronic stage.

We suggest that this model could be useful as a stable experimental model for the study of adult retinal ischemia and it will present an experimental ground for the further investigation of the pathogenesis for human retinal ischemic injuries.

##  Supplemental Information

10.7717/peerj.11665/supp-1 Supplemental Information 1 Raw Data for Figures

10.7717/peerj.11665/supp-2 Supplemental Information 2 Author Checklist

## Additional Information and Declarations

### Data Availability

The following information was supplied regarding data availability: The raw measurements are available in the Supplemental File.
